# Supplementary figures and images for: Severe Myocardial Dysfunction after Non-Ischemic Cardiac Arrest: Effectiveness of Percutaneous Assist Devices
Source: J Clin Med. 2021 Aug 17;10(16):3623. doi: 10.3390/jcm10163623 (PMC8396996; doi:10.3390/jcm10163623)

**Figure S1. Flow delivered by the devices**

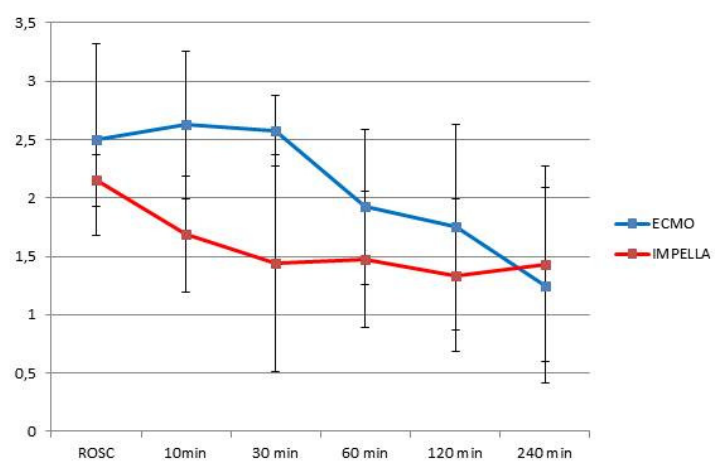

Supplement: Supplementary file 1 [file jcm-10-03623-s001.zip › jcm-1301682-supplementary.pdf]
